# Efficacy of Telemedicine and Telemonitoring in At-Home Monitoring of Patients with COVID-19

**DOI:** 10.3390/jcm10132893

**Published:** 2021-06-29

**Authors:** Emilio Casariego-Vales, Rosa Blanco-López, Benigno Rosón-Calvo, Roi Suárez-Gil, Fernando Santos-Guerra, María José Dobao-Feijoo, Ramón Ares-Rico, Mercedes Bal-Alvaredo, on behalf of the TELEA-COVID Lugo Comanagement Team

**Affiliations:** 1 Internal Medicine Department, Lucus Augusti University Hospital, 27003 Lugo, Spain; roi.suarez.gil@sergas.es (R.S.-G.); Mercedes.Bal.Alvaredo@sergas.es (M.B.-A.); 2 Unidad Administrativa 3B, Lucus Augusti University Hospital, C/Dr. U. Romero, 1, 27003 Lugo, Spain; 3 Day Hospital Nursing, Lucus Augusti University Hospital, 27003 Lugo, Spain; Rosa.Maria.Blanco.Lopez@sergas.es (R.B.-L.); Maria.Jose.Dobao.Feijoo@sergas.es (M.J.D.-F.); 4 Subdirectorate General, Galician Health Service, Ministry of Health, Santiago de Compostela, 15703 A Coruña, Spain; Benigno.Roson.Calvo@sergas.es (B.R.-C.); Fernando.Santos.Guerra@sergas.es (F.S.-G.); 5 Management, Lugo Healthcare Area, A Mariña and Monforte de Lemos, Lucus Augusti University Hospital, 27003 Lugo, Spain; ramon.ares.rico@sergas.es

**Keywords:** COVID-19, telemedicine, mortality

## Abstract

Aim: this work aims to assess if telemedicine and telemonitoring are clinically useful and safe for at-home monitoring of Coronavirus disease 2019 (COVID-19) patients. Methods: This is a retrospective cohort study of all patients diagnosed with COVID-19 in Galicia (Northwestern Spain) between 26 December 2020 and 15 February 2021. The structured, proactive monitoring via telemonitoring (TELEA) of patients considered to be high-risk in the Lugo, A Mariña, and Monforte Healthcare Area (ASLAM) was evaluated compared to other models in the remaining healthcare areas of Galicia. Results: Of the 47,053 COVID-19 patients, 4384 (9.3%) were in ASLAM. Of them, 1187 (27.1%) were monitored via TELEA, and the rest (3197 in ASLAM and 42,669 in the rest of Galicia) were monitored via other methods. Patients monitored in ASLAM via TELEA were older, consulted in the emergency department less frequently (*p* = 0.05), were hospitalized less frequently (*p* < 0.01), had shorter hospital stays (*p* < 0.0001), and had a lower mortality rate in their first hospitalization (*p* = 0.03). No at-home life-threatening emergencies were recorded. Conclusions: these data suggest that, for COVID-19 patients, a care model involving proactive at-home monitoring with telemedicine and telemonitoring is associated with reduced pressure on hospital services and a lower mortality rate.

## 1. Introduction

Coronavirus disease 2019 (COVID-19), the disease caused by the SARS-CoV-2 virus, is characterized by its sudden and widespread dissemination, accelerated clinical progression, and tragic consequences [1,2,3]. Approximately 20% of patients, especially those who are elderly or who have comorbidities, are at risk of progressing to severe forms of the disease, requiring hospitalization [4]. The key to fighting this disease is vaccinating the population [5]. While it is not possible to identify patients with a worse prognosis early, this is very important for personalizing treatment, reducing morbidity and mortality, and assigning appropriate resources in all levels of care [6,7]. Therefore, it is necessary to have monitoring systems that are capable of quickly detecting and treating patients who may need more advanced care in a very brief period of time.

In recent months, telemedicine has proven useful for establishing safe protocols for close at-home monitoring [8], determining the pattern of symptoms [9], or predicting the risk of hospitalization [10] in patients with COVID-19. However, its impact on the course of the disease and its repercussion on healthcare outcomes in the community are not well-known. Since 2015, the Galician Health System (Servicio Galego de Saúde (SERGAS)), in Northwestern Spain, has had a telemedicine tool available as part of the electronic medical record. This tool, called Telemedicina y Telemonitorización Asistencial (TELEA) allows for the at-home monitoring of patients from a “virtual ward” [11]. Since its creation, it has been used for monitoring patients with chronic diseases. Following an adaptation of the tools and procedures in 2020, it is also used in patients with COVID-19 [8]. 

The aim of this study was to analyze the efficacy and safety of the integration of telemedicine and telemonitoring into a system for controlling patients with COVID-19 via at-home monitoring in a well-defined region of Europe.

## 2. Materials and Methods

### 2.1. Study Design

This work is a retrospective cohort study of all individuals diagnosed with COVID-19 via polymerase chain reaction (PCR) and/or antigen detection in nasopharyngeal exudate in the Autonomous Community of Galicia (Northwestern Spain) in the third wave of the COVID-19 pandemic.

For this study, a case was deemed to be part of the third wave if the PCR and/or antigen test had been performed between 26 December 2020 and 15 February 2021 (inclusive). To set these limits, the daily incidence rates were reviewed, and points of inflection were agreed upon. The study setting was the Autonomous Community of Galicia. Its healthcare network provides coverage for nearly all of its 2,701,819 residents [12], and all its epidemiological, microbiological, and clinical information is centralized. In regard to the care model, this network is organized into seven healthcare areas, each of which encompasses hospitals (14 in total) and clinics (at least one in all of its towns). Among them, the Área Sanitaria de Lugo, A Mariña, y Monforte de Lemos (ASLAM) healthcare area provides healthcare coverage to 345,000 residents, and has 3 hospitals and 84 health centers [12].

### 2.2. Telemedicine Tools

SERGAS developed the TELEA tool, which is integrated into the electronic medical record and allows for performing at-home telemedicine and telemonitoring. This instrument/application allows for patients to send messages, questionnaires, clinical parameters or videos to their own medical record [8]. Healthcare personnel can evaluate this information in real time from any point on the network, communicate with the patient, and act accordingly. 

Patient monitoring in the various healthcare areas of Galicia: The TELEA tool was available in all healthcare areas in Galicia. Adapted from an initial design for patients with chronic illnesses, it has been used for the monitoring and control of patients with COVID-19 since 3 March 2020. In ASLAM, teams of professionals were created for the TELEA COVID-19 monitoring program that included medical personnel from the internal medicine and nursing departments. A detailed work protocol that included monitoring criteria and guidelines for the professionals was established and updated in successive versions of the protocol. The protocol version used in this study was that of 15 December 2020. These professionals used pre-established criteria to select patients who would be monitored via this method. The patients not included in TELEA were individually monitored by their respective primary-care physicians who had some form of telemedicine and telemonitoring available to them.

The use of TELEA in patients with COVID-19 is very heterogeneous in the other healthcare areas of Galicia. Though TELEA is used in one way or another in all healthcare areas of Galicia, ASLAM is the only area where this tool is fully integrated into the care model for patients with COVID-19.

### 2.3. Care Model in ASLAM

**Figure 1:**
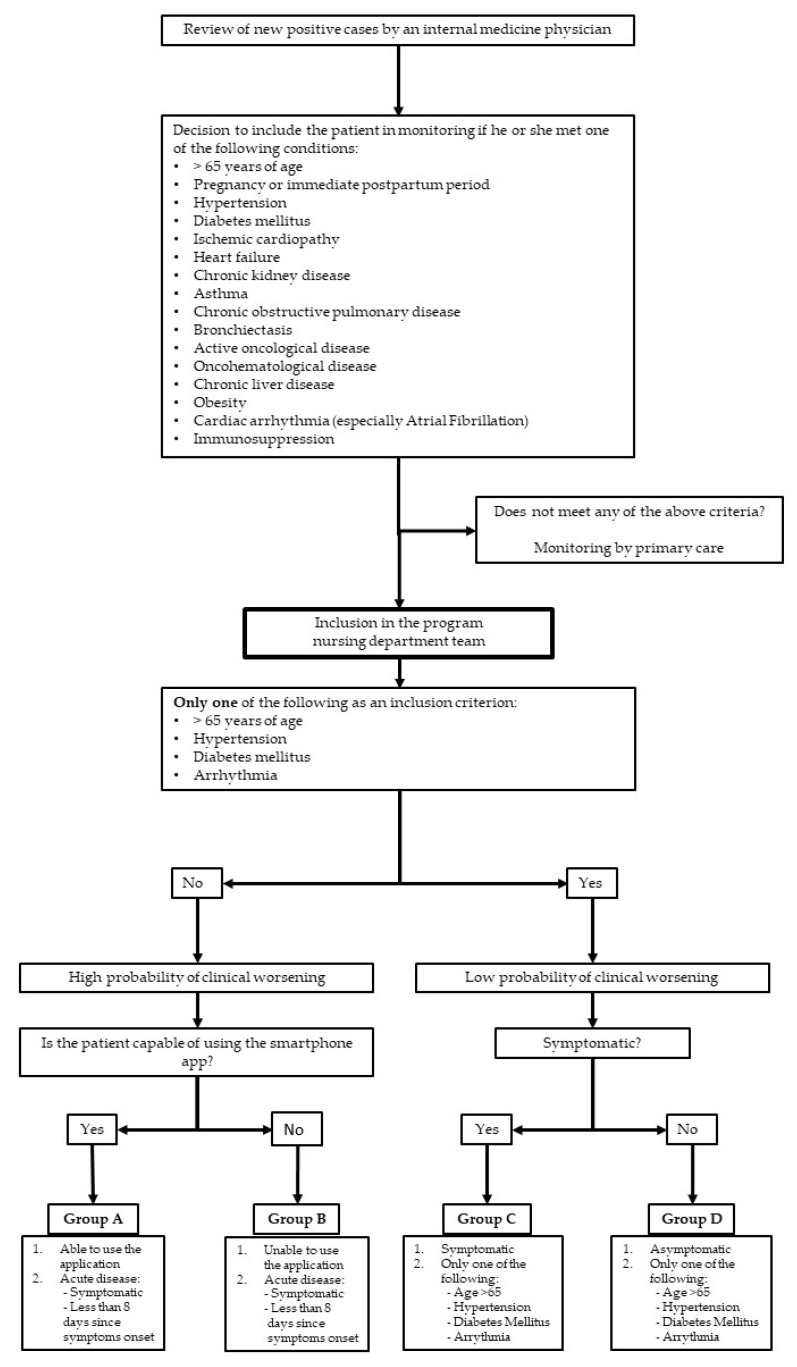
Process for patient selection and inclusion in telemedicine with telemonitoring program (TELEA).

Inclusion of patients in the TELEA program: Every day, the internal-medicine physicians reviewed new positive COVID-19 cases as they were confirmed by the laboratories (Figure 1). For each patient, a comprehensive evaluation of the information available in his or her electronic medical record was performed. Following the review, and using some of the new knowledge acquired in the previous months [8], the inclusion criteria for the TELEA monitoring program were:

(1) Adult patients (older than 14 years of age) with or without symptoms at the time of inclusion.
(2) Reside in a private residence within the ASLAM area and not be hospitalized, even temporarily, or living in a nursing home.
(3) Present with at least one of the following conditions: pregnancy, hypertension, diabetes mellitus, known cardiopathy of any nature, chronic obstructive pulmonary disease, asthma, chronic kidney disease, obesity (body mass index >30), advanced chronic liver disease, or immunosuppression of any origin.
(4) If none of the aforementioned conditions were present, being older than 65 years of age.

Exclusion criteria were:

(1) Patient’s refusal to be monitored via this method.
(2) Impossibility of regular contact with the monitoring team.
(3) Presence of clinical alarm signs on the initial interview that made it necessary to refer the patient to the emergency department and hospitalize the patient at that time.
(4) Patients younger than 65 years of age who did not have any of the conditions listed in point (c) of the inclusion criteria.

Conduct of the TELEA study in ASLAM: The work method and steps of the study were previously reported [8]. A nurse contacted all patients who satisfied the inclusion and exclusion criteria via telephone, explained the aims, characteristics, and conditions of the monitoring (Table 1), and gathered the most relevant clinical data. If alarm signs were noted, the nurse consulted with an internal-medicine physician who evaluated the need for emergency inperson consultation. If there were no alarm signs, oral informed consent for inclusion was requested. If patients accepted, they were offered a mechanism for accessing the application that connected them with their own electronic medical record. Next, they were explained how to enter vital-signs data and how to answer the daily questionnaire. In cases with telemonitoring (monitoring groups A and B), patients were notified that on that same day, and a courier would bring written instructions, a pulse oximeter, and a thermometer to their home, which would be collected at the end of monitoring.

On the basis of data from this first interview, each patient was classified into a monitoring group (A–D) according to their characteristics (Table 1). This classification was dynamic, adapting to each patient’s individual monitoring needs; a patient’s group could be changed at any time during monitoring. The monitoring of all included patients was solely conducted within this program. 

The nursing department team reviewed the periodically received information as per the type of monitoring indicated for that group (Table 1), and proactively contacted patients via telephone, prioritizing according to the received information. In addition, the patient had a contact telephone number that was operational from 8 a.m. to 9:30 p.m. In the event of incidents such as changes in vital signs or clinical condition, as evaluated by the questionnaire [8], the nursing department team contacted the patient. If it was not possible to resolve the problem via telephone, a physician evaluated the situation and decided if it was necessary to transfer the patient to the hospital emergency department. In this case, the physician contacted that department, and explained the reasons why they advised the referral and planned how to continue with at-home monitoring if hospitalization was not deemed necessary.

In addition, all patients who were monitored via TELEA following hospital discharge due to SARS-CoV-2 infection were assigned to monitoring group A. As they had been hospitalized, the data from these patients were not included in this analysis, as per the exclusion criteria.

Discharge from the TELEA Program: A patient could be discharged from monitoring if they met all the following conditions:

(1) At least 10 days had passed since the onset of symptoms.
(2) The patient did not have any symptoms or symptoms were residual.
(3) The patient had been afebrile for at least the last 72 h.
(4) No other problems or medical complications were noted.

TELEA Monitoring Quality Evaluation Criteria

To establish the clinical value of TELEA monitoring, certain criteria were established, as detailed in Table 2.

Nursing department personnel noted any missed monitoring instances and, after re-establishing contact, advised on the need to comply with the scheduled contact for the indicated period of time. At the end of the monitoring period, missed contacts, errors, justifications, etc. were recorded.

Outcome measures: To evaluate the results, two types of outcome measurements were used:

1. Process outcomes: number of patients who completed monitoring, dropouts during the program, and number of days in which there were connection errors or serious technological difficulties.
2. Results outcomes: at-home death, cases referred to the emergency department, hospital admissions, mean length of hospital stay, inhospital mortality, and number of discharges following telemedicine with telemonitoring.

Monitoring of ASLAM patients not included in TELEA: For patients not included in TELEA, their primary-care physicians monitored them via telephone with contact at least once per day in the 10 days following a positive test that determined the presence of the disease. The use of TELEA in this population is inconsistent and not systematic, like in the rest of the areas apart from ASLAM.

### 2.4. Ethical and Legal Aspects

The data were included in a registry approved by the ASLAM Research Ethics Committee. During the initial interview, nursing department personnel explained the conditions of the monitoring, the risks and possible issues, and how the collected data would be processed to each candidate for inclusion. Afterwards, oral informed consent was requested. For this study, the following variables were collected: patient’s place of residence; date of entry into the TELEA program; date of referral to the emergency department and hospitalization, if applicable; and the final result of each of the evaluations. The sources of information included both the data from the monitoring of each patient and records from the various area hospitals. 

### 2.5. Statistical Methods

In creating the patient flowchart and analyzing patient characteristics, the usual descriptive-statistics techniques were used. The chi-squared test was used to compare qualitative variables. After evaluating homoscedasticity, Student’s *t*-test was used to compare two means, and ANOVA was used to compare multiple means. To evaluate the monitoring, the Kaplan–Meier method was used, with 4 March 2021 as the end date. In order to build age-adjusted rates, data gathered from the municipal register of inhabitants as of 1 January 2021 were used [12], and patients were stratified into three age groups (0–59, 60–79, and >80 years). The level of statistical significance was *p* < 0.05. SPSS statistical program v. 18 was used for analysis (SPSS Inc., Chicago, IL, USA).

## 3. Results

Between the start of the pandemic in March 2020 and 15 February 2021, a total of 105,257 individuals were diagnosed with COVID-19 in Galicia. Of them, 47,053 (44.7% of the total) were diagnosed in the third wave (between December 26, 2020 and February 15, 2021). The distribution of these third-wave patients according to healthcare areas was as follows: 4384 (9.3%) were diagnosed in ASLAM, and the remaining 42,669 in the six other areas. For the patients in ASLAM, a physician evaluated the electronic medical records in the initial hours following disease detection. A total of 1191 patients were considered to meet the inclusion criteria and were candidates for monitoring via TELEA. The remaining 3193 patients did not meet the criteria; therefore, monitoring was conducted by their primary-care physician. Among the latter, there were 215 (6.7%) patients who resided in nursing homes. This subgroup of patients had a special monitoring program available directly in their centers, and, for the purposes of this study, their data were analyzed together with data from patients monitored via primary care.

Following initial contact, only 4 (0.3%) patients rejected monitoring via TELEA. Therefore, 1187 (27.1%) patients in the healthcare area were monitored via the TELEA program, and 3197 (72.9%) were monitored by their primary-care physicians. The characteristics of patients monitored via the TELEA program are shown in Table 3.

**Figure 2:**
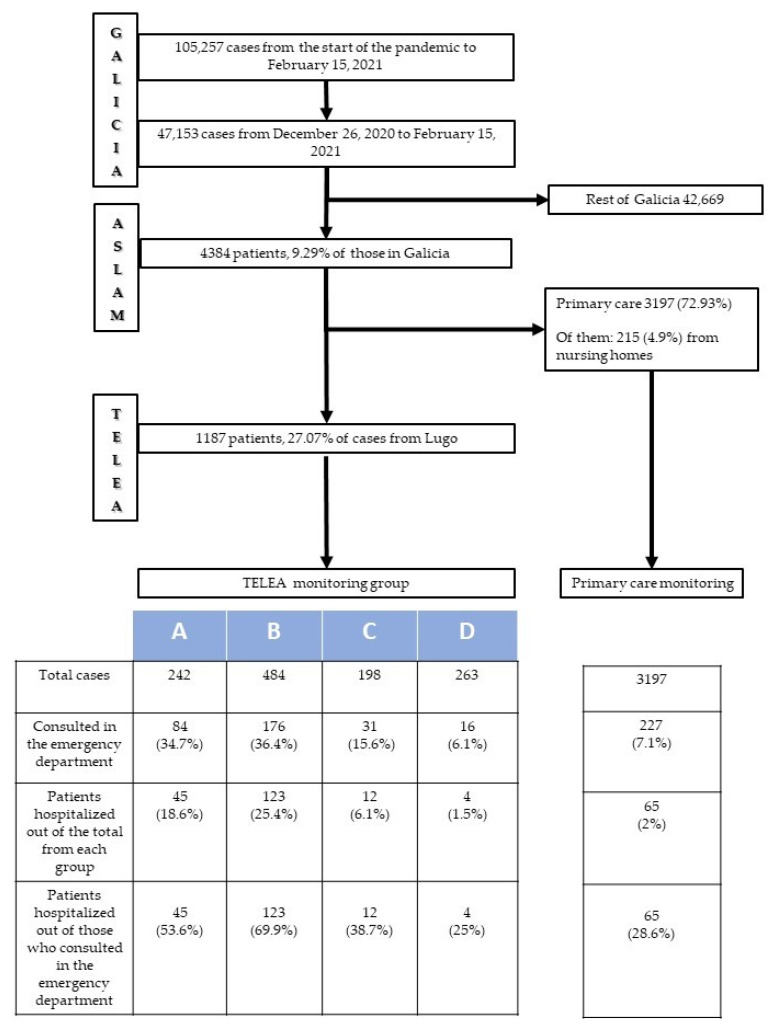
Diagram for COVID-19 patients in ASLAM: Hospital requirements.

Figure 2 shows the different pathways for patients from ASLAM according to the type of monitoring program to which each was assigned. Patients monitored in the various TELEA monitoring groups (A, B, C, D) had a greater number of consultations in hospital emergency departments (*p* < 0.0001) and a greater number of hospitalizations (*p* < 0.0001) than those monitored by primary-care physicians. Likewise, patients assigned to TELEA programs A and B required both a greater number of emergency department visits (*p* < 0.0001) and hospitalizations (*p* < 0.0001) than those included in lower-intensity groups C and D. Indeed, those in lower-intensity groups had emergency department consultation and hospitalization figures that were very similar to patients monitored via primary care. 

When comparing the characteristics of patients from ASLAM to those of patients from the rest of Galicia, no differences according to sex were noted (53.4% and 53.6% were women; *p* = 0.6). The age of patients in ASLAM was significantly higher than those in the rest of Galicia: 46.34 (SD 24.4) vs. 44.98 (SD 26.28) for total cases, 69.53 (SD 17.47) vs. 68.29 (SD 17.43) for hospitalizations, and 83.87 (SD 8.74) vs. 81.51 (SD 10.16) for those who died in the hospital (*p* = 0.0001, 0.007, and 0.001, respectively).

The achieved healthcare outcomes with this monitoring model compared to those from other regions of Galicia are shown in Table 4. Adjusted for age, there were 1.3 admissions per 1000 inhabitants in ASLAM, whereas the ratio in the rest of Galicia was 1.8/1000 inhabitants. Patients in monitoring in ASLAM came to the hospital emergency department less (*p* = 0.05), required hospitalization in lower numbers (*p* < 0.01), and had shorter hospital stays (*p* < 0.0001). Lastly, significantly fewer patients died in their first hospitalization in ASLAM compared to the rest of Galicia (*p* = 0.03). In fact, after adjusting for age, 17.6 deaths were recorded per 100,000 inhabitants in ASLAM whereas the ratio in the rest of Galicia was 29.2/100,000 inhabitants.

**Figure 3:**
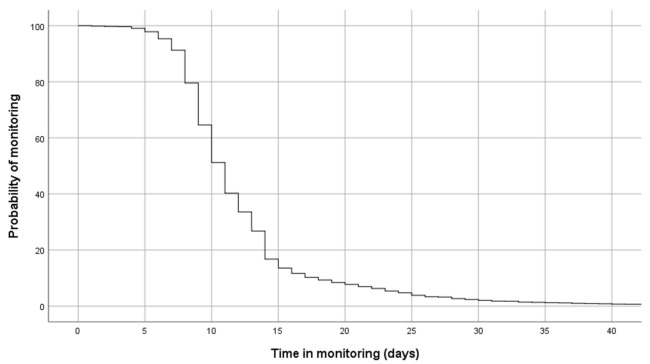
Probability of continuing in monitoring via TELEA from date of inclusion.

Figure 3 shows the monitoring periods for the program. The median time of monitoring was 11 days (95% CI 10.8–11.1 days), and the probability of remaining in the program after 20 days was 9.2%. The number of contacts between TELEA and the patients varied widely according to the number of days of monitoring or the need for hospitalization. Patients who presented with persistent symptoms required longer monitoring periods. On the other hand, individuals who had more severe disease were generally referred to the hospital within a brief period of time (mean monitoring time of 3.5 days, SD 3.1). The established protocols were appropriately complied with in 802 cases (67.6%), complied with inconsistently but in a way that was considered sufficiently valid for clinical management in 201 cases (16.9%), and were not appropriately complied with in 184 cases (15.5%). In these last two groups, the process was not correctly followed in 27.8% of cases due to technical reasons or the incorrect use of the technology. 

During monitoring, four at-home deaths were recorded (0.3%). All four were patients with very advanced chronic diseases (two had Alzheimer’s disease, and two had a metastatic disease: gastric and lung tumors) who had a poor baseline condition and whose families preferred at-home treatment. On the other hand, no at-home life-threatening emergencies were recorded. All emergency transfers to the hospital were conducted using ordinary means, and it was not necessary to send life-support teams to any home. 

## 4. Discussion

This study shows that the integration of telemedicine with telemonitoring used proactively in a structured care model for patients with COVID-19 detected at the time of diagnosis and quarantined at home is associated with a frank decrease in hospitalizations, mean length of hospital stay, and mortality. Furthermore, these results suggest that the at-home care of these patients via telemedicine is safe.

Though the usefulness of telemedicine in the management of chronic diseases is known [13,14], its role in patients with acute infectious diseases has not been as extensively analyzed [15,16]. In patients with COVID-19, it is useful for determining risk of hospitalization [10], describing the disease’s clinical course [9], and evaluating at-home treatment indications [17]. It is an appropriate system for patient monitoring [8,18]. Our data reinforce the idea that telemedicine with telemonitoring tools integrated into habitual care are well-accepted in the home setting, and are effective and safe for the at-home monitoring of COVID-19 patients. First, our model includes the rapid identification of candidates for inclusion in the program and the appropriate selection of those who have a greater probability of requiring hospital resources. In this regard, although TELEA monitored 27.1% of patients, 73.9% of all hospital admissions due to COVID-19 in that period came from this group. Second, the stratification of patients into risk groups that entail different types of monitoring is useful. Figure 2 shows that up to 2 of every 3 admissions were for patients assigned to monitoring groups A and B, which were for patients considered to be at greater risk. This suggests that the criteria for selection and distribution into monitoring groups were appropriate. The close monitoring of higher-risk patients significantly reduced hospital admissions in this group to the extent that it improved the study’s overall outcomes. In addition, concentrating greater monitoring efforts on higher-risk patients also allows for the simultaneous management of a greater number of patients. Lastly, the operation of a virtual ward, including control systems and protocols that governed the actions taken, did not yield any serious problems. In fact, for the four patients who died, prior agreement had been reached not to transfer the patient to the hospital. All of this suggests that this care model allows for effective at-home surveillance and a safe hospital referral for the most severe cases, which helps to optimize the care model as a whole. 

Our healthcare outcomes are significantly better than those in our surroundings. When evaluating these outcomes, the healthcare network of which all study’s centers form part is quite homogeneous, and all centers have comparable resources and availability. There are also no notable differences among the patients attended to from one center to another. Lastly, in the third wave of infections, vaccination in nursing homes (not included in the TELEA program) was very advanced. Therefore, the disease had very few repercussions on nursing homes, and there were few hospitalizations of their residents. Thus, differences in outcomes must largely be attributed to the organization of healthcare in response to the disease. In ASLAM, TELEA allowed for at-home control by physicians and nurses with experience in this type of consultation and in COVID-19 monitoring. This experience is invaluable when it comes to identifying disease progression and making the right decisions at the right time [19]. It likely contributed to reducing emergency-department consultations and managing the flow of hospital admissions. Although our study did not allow for us to definitively determine so, it is possible that this work method made it possible for patients to be admitted at earlier stages of the disease, and for treatment to be initiated earlier, which is associated with better prognosis [20,21]. On the other hand, the fact that there was postdischarge surveillance probably led to earlier discharges and could have contributed to reducing the mean length of stay. As a whole, surveillance and decision making by experienced personnel of patients considered to be at greater risk contributed to reducing the most severe consequences of this disease.

The use of telemedicine obligated both healthcare personnel and patients to acquire new skills [22]. In our case, part of the personnel had experience using the system in programs for chronic patients. This facilitated the incorporation of new professionals, who were trained in the previous waves; they learned relatively fast. On the other hand, this new technology and method of communication may not be feasible for elderly or more vulnerable people [23]. To mitigate these problems, each patient’s difficulties were evaluated in the initial conversation. With this information, they were assigned to the various monitoring groups (A, B, C, D) according to their clinical condition and technology skills. For example, group B included patients who did not feel capable of independently participating in telemonitoring and required remote assistance by healthcare personnel. Our understanding is that remote support by healthcare personnel, though it takes time and requires more professionals, improves service and has evident repercussions on care.

The proposed telemedicine system had a high rate of acceptance and adherence. In fact, only an anecdotal number of patients rejected monitoring via TELEA. Among those who participated, 2 out of every 3 patients were considered to have received adequate monitoring, and monitoring was deemed insufficient in just 15.5% of cases. The good reception of the program is likely a result of to various circumstances. It was undoubtedly related to the patients’ situation, as they were locked down at home with a potentially severe disease. In this scenario, being able to communicate quickly and easily with a monitoring team and send information on one’s condition with the certainty of obtaining a rapid response is highly valuable to the patient. In addition, the fact that monitoring was brief but able to be extended according to the patient’s symptoms likely increased acceptance of and trust in the system. It was clear to patients that it was not the tools, such as the pulse oximeters, but rather the quality of clinical care that protected their lives. Therefore, proactive control, good training, and the experience of the healthcare personnel in managing the process were also key [24]. Appropriate training on the management of this disease, and on the use and particularities of the tool or the standardized actions taken by professionals are very important aspects for achieving a high level of adherence and good quality care. 

This study must be evaluated taking into account its strengths and weaknesses. Its main strength is that it includes data from all diagnosed COVID-19 cases in a specific region and in a well-defined time period. Given that the information is gathered and verified for clinical use in real time, the data are robust and there were minimal missing values. There are also certain limitations. First, care via TELEA was only conducted in one of the autonomous community’s seven healthcare areas. Although this provides highly homogeneous information, it is necessary to replicate the process, and confirm the outcomes with other teams and in other areas. Second, the chosen time period can be considered brief and did not encompass the entire duration of the pandemic. In regard to the selected period, our understanding is that, given the study’s aim, it was more appropriate to analyze a period in which the healthcare system was significantly overwhelmed, and this was the most intense wave in our autonomous community. There were other reasons for selecting this period, such as having well-trained, experienced personnel who had honed their skills in the first and second waves, and the minimal number of patients in nursing homes included, as their particular characteristics could have skewed the data. On the whole, with all of these choices, a better evaluation of the results was possible. Third, it was not possible to obtain some data, such as the date of symptom onset, which was not always easy to determine in the initial interview. Lastly, we analyzed deaths that had occurred during hospitalization and not those that had occurred following discharge. This was decided upon given that the study’s aim was to determine the effect of TELEA on the management of patients prior to a hospitalization. Nevertheless, it is necessary to conduct further studies to establish long-term prognosis. 

## 5. Conclusions

Including proactive at-home monitoring with telemedicine and telemonitoring within the care model for the control of the COVID-19 pandemic led to a reduction in the overloading of hospitals and a lower mortality rate. Though our data suggest that this care model is highly effective, additional studies are necessary to confirm these findings in other areas with different conditions.

## Figures and Tables

**Table 1:** Actions by different participants in the process according to proposed type of monitoring at the time of patient inclusion.

**Nurse Actions**	- Initial telephone contact with the patient - Identification and presentation - Justification for the call - Explanation of monitoring			
	- Determination of monitoring group based on a survey on clinical progress, patient characteristics, and risk of clinical worsening - Reporting of incidents found on the initial evaluation (degree of obesity, postpartum status, social problems, or other) to the physician			
	**Monitoring Groups**			
	**A**	**B**	**C**	**D**
	Review temperature and O_2_ saturation vital signs three times per day. Review morning-symptoms survey. Telephone contact with the patient once per day.	Telephone contact with the patient every day at 8 a.m. and 8 p.m. Symptoms survey. Temperature and O_2_ saturation check.	Telephone contact with the patient every other day. Symptoms survey and temperature check.	On-demand contact: The patient reports any change in their clinical condition to the nurse.
	Report incidents to the physician if necessary			
**Patient Actions**	Check vital signs (temperature and O_2_ saturation) 8–9 a.m., 2–3 p.m., 7–8 p.m. Respond to symptoms survey (8–9 a.m.). Enter vital signs data into the TELEA app.	Check vital signs (temperature and O_2_ saturation).	Check temperature every 8 h.	Check temperature every 8 h.
	Report alarm signs and symptoms to the telemonitoring nursing team.			
**Physician Actions**	- Contact patient to resolve alerts reported by the nursing department - Refer patient to the emergency department if necessary - Report referral to the emergency department - Discharge patient			

**Table 2:** Quality criteria in the monitoring by TELEA.

Monitoring appropriately complied with if: At least 90% of the planned monitoring instances or contacts were conducted as scheduled. Fewer than three consecutive monitoring instances not conducted. After not conducting one of the planned monitoring instances, the patient responded to a telephone call from the personnel and justified the delay. Monitoring complied with inconsistently, but clinically useful if: At least 80% of the planned monitoring instances or contacts were conducted as scheduled. Fewer than three consecutive monitoring instances not conducted. After not conducting one of the planned monitoring instances, the patient responded to a telephone call from the personnel and justified the delay. Monitoring not appropriately complied with and not clinically useful if: Less than 80% of the planned monitoring instances or contacts were conducted as scheduled. Three or more consecutive monitoring instances not conducted. After not conducting one of the planned monitoring instances, the patient did not respond to a telephone call, it was noted that they had not complied with the rules (for example, not remaining isolated) or they did not justify the delay.

**Table 3:** General characteristics of patients monitored with telemedicine and telemonitoring in ASLAM.

	Patients and Monitoring (*N* = 1187)
Sex (male)	596 (50.3%)
Age	
Mean age; SD	65.6; 15.9 years
Range	15–99
Age groups	
18–40	92 (7.7%)
41–50	120 (10.1%)
51–60	194 (16.3%)
61–70	285 (24.1%)
71–80	279 (23.5%)
81–90	182 (15.4%)
≥91	35 (2.9%)
Time between symptom onset and start of telemedicine: mean; SD	1.9; 2.9 days
Hypertension	658 (55.5%)
Diabetes mellitus	266 (22.4%)
Obesity (BMI > 30)	178 (15%)
Cardiac arrhythmia	92 (7.8%)
Immunosuppression	82 (6.9%)
Nonhematologic neoplasm	76 (6.4%)
Ischemic cardiopathy	68 (5.7%)
Chronic obstructive pulmonary disease	58 (4.9%)
Asthma	56 (4.7%)
Heart failure	36 (3.0%)
Chronic kidney disease	26 (2.2%)
Chronic liver disease	21 (1.8%)
Hematologic disease	16 (1.3%)
Pregnancy	14 (1.2%)

**Table 4:** Healthcare outcomes of all patients with COVID-19 in ASLAM versus those registered in the rest of Galicia.

	Cases	First Emergency Department Visit	Hospital Admission	Length of Stay in Days (Mean, SD)	Inhospital Deaths
ASLAM	4384 (9.3%)	552 (12.6%)	262 (47.5%)	8.16 (6.3)	30 (11.5%)
Rest of Galicia	42,669 (90.7%)	5827 (13.7%)	3095 (53.1%)	10.48 (7.9)	511 (16.5%)
*p*		0.05	0.001	0.0001	0.03

## Data Availability

The data presented in this study are available upon request from the corresponding author, and are not publicly available due to patient data protection.

## References

[1] Liu S.-L., Saif L. (2020). Emerging Viruses without Borders: The Wuhan Coronavirus. Viruses.

[2] Guan W.J., Ni Z.Y., Hu Y., Liang W.H., Ou C.Q., He J.X., Liu L., Shan H., Lei C.L., Hui D.S.C. (2020). Clinical Characteristics of Coronavirus Disease 2019 in China. N. Engl. J. Med..

[3] Wu Z., McGoogan J.M. (2020). Characteristics of and important lessons from the coronavirus disease 2019 (COVID-19) outbreak in China: Summary of a report of 72 314 cases from the Chinese Center for Disease Control and Prevention. JAMA.

[4] Richardson S., Hirsch J.S., Narasimhan M., Crawford J.M., McGinn T., Davidson K.W., Barnaby D.P., Becker L.B., Chelico J.D., Cohen S.L. (2020). Presenting characteristics, comorbidities, and outcomes among 5700 patients hospitalized with COVID-19 in the New York City Area. JAMA.

[5] Hossain K., Hassanzadeganroudsari M., Feehan J., Apostolopoulos V. (2021). COVID-19 Vaccines in the Pipeline, Are Antibodies Adequate?. Vaccines.

[6] Marin B.G., Aghagoli G., Lavine K., Yang L., Siff E.J., Chiang S.S., Salazar-Mather T.P., Dumenco L., Savaria M.C., Aung S.N. (2021). Predictors of COVID-19 severity: A literature review. Rev. Med. Virol..

[7] Soy M., Keser G., Atagündüz P., Tabak F., Atagündüz I., Kayhan S. (2020). Cytokine storm in COVID-19: Pathogenesis and overview of anti-inflammatory agents used in treatment. Clin. Rheumatol..

[8] Martínez-García M., Bal-Alvarado M., Santos Guerra F., Ares-Rico R., Suárez-Gil R., Rodríguez-Álvarez A., Pérez-López A., Casariego-Vales E., en nombre del Equipo de Seguimiento Compartido TELEA-COVID Lugo, Equipo TELEA COVID-19 (Lugo) (2020). Monitoring of COVID-19 patients by telemedicine with telemonitoring. Rev. Clin. Esp..

[9] O’Keefe J.B., Tong E.J., O’Keefe G.D., Tong D.C. (2021). Description of symptom course in a telemedicine monitoring clinic for acute symptomatic COVID-19: A retrospective cohort study. BMJ Open.

[10] O’Keefe J.B., Tong E.J., Taylor T.H., O’Keefe G.A.D., Tong D.C. (2021). Use of a Telemedicine Risk Assessment Tool to Predict the Risk of Hospitalization of 496 Outpatients With COVID-19: Retrospective Analysis. JMIR Public Health Surveill..

[11] Casariego E., Guerra F., Cerqueiro J.M., Rosón B., Chaos P., Guerrero H. Telemedicina en ICC: Eficacia clínica y valor para el paciente. XXI Congreso Nacional de Hospitales y Gestión Sanitaria. Premio Patient Journey. Santiago de C, 8de mayo de 2019 [consultado 16 Abr 2020]. https://www.21congresohospitales.org/media/attachments/2019/05/09/programacompleto09052019.pdf.

[12] Instituto Nacional de Estadística Cifras oficiales de población. https://www.ine.es/jaxiT3/Datos.htm?t=2853.

[13] Batsis J.A., DiMilia P.R., Bs L.M.S., Fortuna K.L., Kennedy M.A., Blunt H., Bagley P.J., Brooks J., Brooks E., Kim S.Y. (2019). Effectiveness of ambulatory telemedicine care in older adults: A systematic review. J. Am. Geriatr. Soc..

[14] Zhu Y., Gu X., Xu C. (2020). Effectiveness of telemedicine systems for adults with heart failure: A meta-analysis of randomized con-trolled trials. Heart Fail. Rev..

[15] Young J.D., Abdel-Massih R., Herchline T., McCurdy L., Moyer K.J., Scott J.D., Wood B.R., Siddiqui J. (2019). Infectious Diseases Society of America Posi-tion Statement on telehealth and telemedicine as applied to the practice of infectious diseases. Clin. Infect. Dis..

[16] Parmar P., Mackie D., Varghese S., Cooper C. (2014). Use of telemedicine technologies in the management of infectious diseases: A review. Clin. Infect. Dis..

[17] O’Keefe J.B., Newsom L.C., Taylor T.H. (2021). A Survey of Provider-Reported Use and Perceived Effectiveness of Medications for Symptom Management in Telemedicine and Outpatient Visits for Mild COVID-19. Infect. Dis. Ther..

[18] Orrange S., Patel A., Mack W.J., Cassetta J. (2021). Patient satisfaction and trust in telemedicine during the COVID-19 pandemic. JMIR Hum. Factors.

[19] Thornton J. (2020). The “virtual wards” supporting patients with covid-19 in the community. BMJ.

[20] Fadel R., Morrison A., Vahia A., Smith Z.R., Chaudhry Z., Bhargava P., Miller J., Kenney R.M., Alangaden G., Ramesh M.S. (2020). Early Short-Course Corticosteroids in Hospitalized Patients with COVID-19. Clin. Infect. Dis..

[21] Sundararaj Stanleyraj J., Sethuraman N., Gupta R., Thiruvoth S., Gupta M., Ryo A. (2021). Treating COVID-19: Are we missing out the window of opportunity?. J. Antimicrob. Chemother..

[22] Iyengar K., Jain V.K., Vaishya R. (2020). Pitfalls in telemedicine consultations in the era of COVID 19 and how to avoid them. Diabetes Metab. Syndr. Clin. Res. Rev..

[23] Ezzat A., Sood H., Holt J., Ahmed H., Komorowski M. (2021). COVID-19: Are the elderly prepared for virtual healthcare?. BMJ Health Care Inform..

[24] Greenhalgh T., Knight M., Inada-Kim M., Fulop N.J., Leach J., Vindrola-Padros C. (2021). Remote management of covid-19 using home pulse oximetry and virtual ward support. BMJ.

